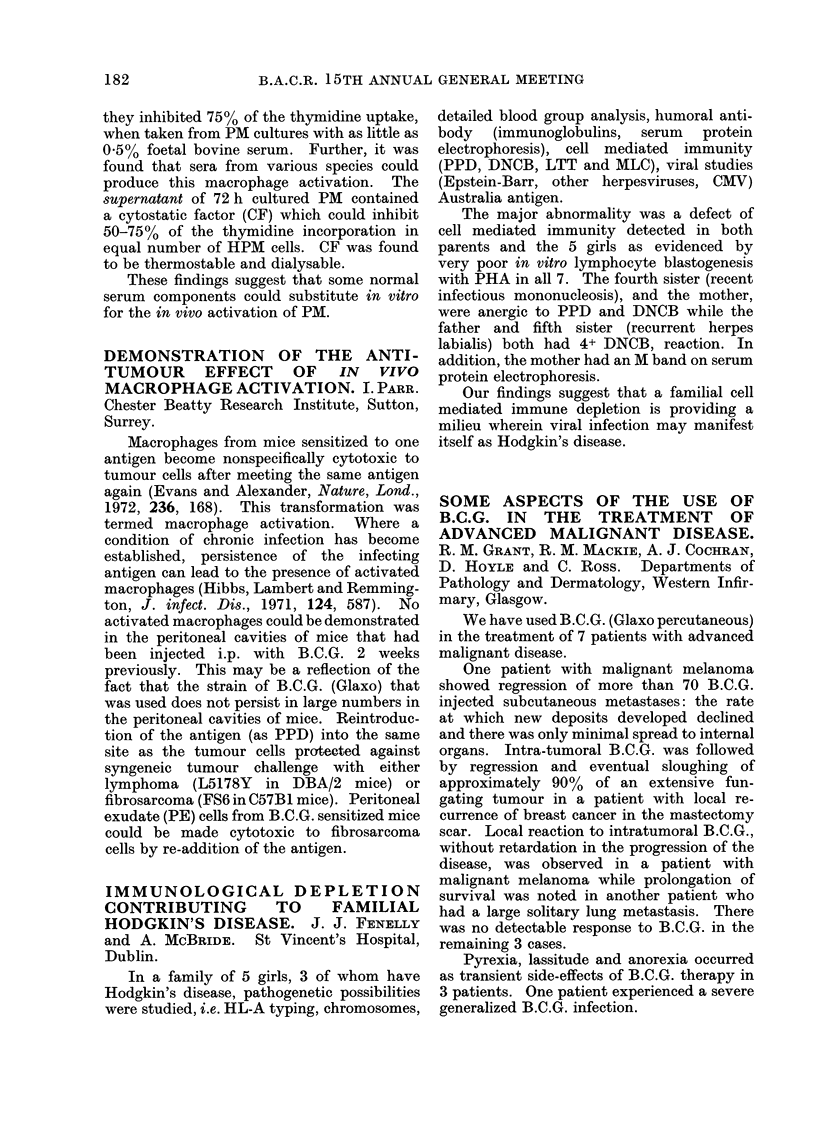# Proceedings: Some aspects of the use of B.C.G. in the treatment of advanced malignant disease.

**DOI:** 10.1038/bjc.1974.164

**Published:** 1974-08

**Authors:** R. M. Grant, R. M. Mackie, A. J. Cochran, D. Hoyle, C. Ross


### SOME ASPECTS OF THE USE OF B.C.G. IN THE TREATMENT OF ADVANCED MALIGNANT DISEASE.

R. M. GRANT, R. M. MACKIE, A. J. COCHRAN, D. HOYLE and C. ROSS. Departments of Pathology and Dermatology, Western Infirmary, Glasgow.

We have used B.C.G. (Glaxo percutaneous) in the treatment of 7 patients with advanced malignant disease.

One patient with malignant melanoma showed regression of more than 70 B.C.G. injected subcutaneous metastases: the rate at which new deposits developed declined and there was only minimal spread to internal organs. Intra-tumoral B.C.G. was followed by regression and eventual sloughing of approximately 90% of an extensive fungating tumour in a patient with local recurrence of breast cancer in the mastectomy scar. Local reaction to intratumoral B.C.G., without retardation in the progression of the disease, was observed in a patient with malignant melanoma while prolongation of survival was noted in another patient who had a large solitary lung metastasis. There was no detectable response to B.C.G. in the remaining 3 cases.

Pyrexia, lassitude and anorexia occurred as transient side-effects of B.C.G. therapy in 3 patients. One patient experienced a severe generalized B.C.G. infection.